# Quality-of-life and beliefs about medication in relation to a therapy adherence intervention in resistant hypertension: the Resistant HYpertension: MEasure to ReaCh Targets trial

**DOI:** 10.1097/HJH.0000000000003780

**Published:** 2024-05-23

**Authors:** Victor J.M. Zeijen, Laura E.J. Peeters, Azra Asman, Eric Boersma, Emma K. Massey, Liset van Dijk, Joost Daemen, Jorie Versmissen

**Affiliations:** a Department of Cardiology; b Department of Internal Medicine; c Department of Hospital Pharmacy, Erasmus University Medical Center, Rotterdam; d Netherlands Institute for Health Services Research (NIVEL), Utrecht; e Department of PharmacoTherapy, Epidemiology and Economics (PTEE), Groningen Research Institute of Pharmacy, Faculty of Science and Engineering, University of Groningen, Groningen, the Netherlands

**Keywords:** antihypertensive agents, hypertension, patient compliance, patient-reported outcome measures, quality of life, surveys and questionnaires

## Abstract

**Objective:**

To assess the impact of personalized feedback on therapy adherence testing results on quality of life and beliefs about medication in patients with resistant hypertension, as well as to identify patient-oriented predictors of therapy adherence.

**Methods:**

This study was a prespecified post hoc analysis of the multicenter randomized controlled trial Resistant HYpertension: MEasure to ReaCh Targets (RHYME-RCT). Patients were randomized to a personalized feedback conversation on measured antihypertensive drug levels additional to standard-of-care, or standard-of-care only. The primary outcomes consisted of EuroQol EQ-5D-5L and Beliefs about Medicine Questionnaire (BMQ) scores at 12 months.

**Results:**

A total of 56 patients with median age 61.5 [25th–75th percentile: 55.8–69.3] years (21.4% women) were included. Mean blood pressure ±SD was 149.8/84.1 ± 14.9/13.8 mmHg while being on a median of 5.6 [4.8–7.3] defined daily dosages (DDD) of antihypertensive drugs. At 12 months, no differences were observed in EQ-5D-5L index (0.81 [0.69–0.89] vs. 0.89 [0.73–1.00]; *P* = 0.18) and visual analogue scale score on general patient-perceived health (70 [60–80] vs. 70 [60–82]; *P* = 0.53) between the intervention-arm and the standard-of-care only-arm. Likewise, individual EQ-5D-5L domain scores and BMQ scores did not differ between both arms. Irrespective of the intervention, independent positive predictors of the percentage adherence were patient age, EQ-5D-5L index score, BMQ-specific necessity score and concern score, whereas the total number of drugs prescribed was a negative predictor.

**Conclusion:**

Within this prespecified subanalysis of the randomized RHYME-RCT trial, implementation of a personalized feedback conversation targeting therapy adherence did not improve health-related quality-of-life and beliefs about medication in patients with resistant hypertension.

## INTRODUCTION

Hypertension is a common cardiovascular risk factor and the leading cause for disability-adjusted life-years [1,2]. Whereas antihypertensive drug therapy demonstrated to reduce cardiovascular risk, blood pressure (BP) remains uncontrolled in 60% of the patients [3–6]. Within hypertensive patients, approximately 10% suffers from resistant hypertension [7]. Nonadherence to antihypertensive drugs has been identified as a major contributor to (suspected) resistant hypertension and is an independent risk factor for cardiovascular adverse events [8,9].

Recently, the dried-blood-spot sampling method was introduced as a reliable and convenient method to measure adherence to antihypertensive drugs [10]. The Resistant HYpertension: MEasure to ReaCh Targets (RHYME-RCT) trial, of which the primary outcome has been published previously, demonstrated a positive effect of a personalized feedback conversation about dried-blood-spot adherence testing on adherence to antihypertensive drugs as compared with standard-of-care only [11,12]. However, this intervention did not improve BP levels or resistant hypertension [12].

Patient-reported outcome measures could further guide the positioning of therapy adherence interventions in the treatment of hypertension. Specifically, health-related quality-of-life (HRQOL) and patients’ beliefs about antihypertensive medication could provide insights into recent adherence intervention studies, such as the RHYME-RCT study. Cross-sectional data showed that lower therapy adherence is associated with impaired HRQOL [13–15]. Up until to date, a paucity of prospective data is available on the effect of therapy adherence interventions on HRQOL [16–18]. In parallel, beliefs about medication are correlated to therapy adherence, as beliefs about harmful drug effects, overuse of drugs and drug inefficacy increased the risk of nonadherence [19–22]. A single intervention study demonstrated that an adherence intervention reduced patient concerns and harmful beliefs with regard to antihypertensive medication [23].

The primary aim of the current study was to investigate the effect of a personalized feedback conversation about therapy adherence testing results on HRQOL and beliefs about medication in resistant hypertension patients [11]. The secondary aim was to identify patient-oriented predictors of adherence to antihypertensive drugs.

## METHODS

### Study design

This study was a prespecified subanalysis of the RHYME-RCT study, which was a single-blinded multicenter randomized controlled trial in which patients were 1 : 1 randomized to receive personalized feedback based on therapy adherence testing results in addition to standard-of-care versus standard-of-care only (ICTRP/Dutch Trial registry: NTR6914) [11].

### Study population

The inclusion criteria and exclusion criteria of the study have been described previously [11]. In short, adult patients with resistant hypertension who were prescribed at least two dried-blood-spot detectable antihypertensive drugs were included. Resistant hypertension was defined as (1) office SBP greater than 140 mmHg and/or DBP greater than 90 mmHg, or daytime ambulatory SBP greater than 135 mmHg and/or DBP greater than 85 mmHg, while (2) prescribed at least three antihypertensive drug classes including a diuretic, or at least four antihypertensive drug classes, all in the highest tolerable dosages. Patients with an estimated glomerular filtration rate (eGFR) less than 15 ml/min/m^2^ or no previous adequate diagnostic testing to exclude secondary causes of hypertension were excluded. All patients provided written informed consent, and the study was approved by the local ethical committee (MEC-2018–027).

### Intervention and standard-of-care

All patients received standard-of-care as per local routine practice and underwent 24 h ambulatory BP measurements (or automated office BP measurements, only per patient preference from the 3-month visit onwards) and therapy adherence testing (drug concentrations in blood determined by dried-blood-spot sampling) at baseline and 3, 6 and 12 months of follow-up. In the standard-of-care arm, patients and healthcare providers were blinded for adherence testing results throughout the study. In the intervention arm, in addition to standard-of-care, the results of the adherence testing were discussed with the patient during a comprehensive personalized feedback conversation with their healthcare provider at baseline (post randomization) and at 3 months [11].

### Patient questionnaires

All patients were invited to complete questionnaires on HRQOL and beliefs about medicine at baseline (prerandomization) and 12-month follow-up.

For the assessment of HRQOL, the five-level EuroQol 5D-5L (EQ-5D-5L) questionnaire was utilized involving five different dimensions (mobility, self-care, usual activities, pain/discomfort and anxiety/depression) and a global visual analogue scale (VAS) score on general patient-perceived health [24]. EQ-5D-5L index scores, based on the five different dimensions, were calculated using the Dutch tariff [25].

For assessment of patient beliefs about the use of medication, the 18-question Beliefs About Medicine (BMQ) questionnaire was adopted [26]. In this questionnaire, the ‘general’ section addressed concerns about harmful drug effects and overuse of medication by healthcare providers, in general. The ‘specific’ section focused on the necessity of medication and concerns about the effectiveness of medication within the individual patient. Patient attitude was classified based on accordance or discordance between the specific necessity and concern scores (Supplementary Methods 1).

### Outcomes

The primary outcomes of this study were the EQ-5D-5L index score and the general patient-perceived health VAS score at 12 months post randomization. Secondary outcomes were the individual dimensions of the EQ-5D-5L questionnaire (mobility, self-care, usual activities, pain/discomfort, and anxiety/depression), the BMQ-General scores (harm, overuse) and BMQ-Specific scores (necessity, concern, and attitude) at 12 months post randomization.

Second, an exploratory analysis on potential predictors of the level of therapy adherence was performed, out of a predefined set of covariates (age, sex, EQ-5D-5L index and VAS score, BMQ general and specific scores, the total number of drugs and the number of antihypertensive drugs). Therapy adherence was assessed using dried-blood-spot sampling at five different time points (baseline and 3, 6, 9, and 12 month follow-up) and was calculated as the percentage of detectable drugs out of all drugs prescribed. Antihypertensive drugs for which dried-blood-spot sampling has not yet been validated were excluded from this calculation [27].

The current study deviated from the prespecified protocol of the RHYME RCT study as no cost-utility and cost-effectiveness analyses were performed [11]. Given that the intervention studied was not effective in the improvement of RH (main outcome of the study), these analyses were considered futile and were, therefore, discontinued [12].

### Statistical analysis

Continuous variables were reported as mean ± standard deviation (SD) or median [25th–75th percentile] for variables with a normal or a skewed distribution, respectively. Skewness was assessed based on quantile–quantile plots and the Shapiro–Wilk test. Categorical variables were reported as counts (percentages).

Between-group differences in continuous variables, including the primary outcomes, were analyzed using the unpaired *t* test or Mann–Whitney *U* test for variables with a normal or a skewed distribution, respectively. Within-group differences in continuous variables were analyzed using the paired *t* test or Wilcoxon signed rank test for variables with a normal or a skewed distribution, respectively. Between-group comparison of categorical variables was performed using Fisher's exact test.

Predictors of adherence were studied using linear mixed-effects models. The percentage of drugs detected using dried-blood-spot sampling was implemented as the outcome variable. Fixed effects were considered for all determinants, except the presence of multiple measurements per patient, which was modelled as random effect. Predictors were assessed using a multivariable model including a predefined set of covariates. Regression coefficients [including the 95% confidence intervals (CI) and corresponding *P* values] were reported for the crude effect of each predictor.

The current study was not statistically powered for its primary outcomes, as it was a predefined secondary analysis of the RHYME-RCT trial [11]. Unless stated otherwise, two-tailed *P* values less than 0.05 were considered statistically significant. Analyses were performed using R 4.2.1 using the packages ‘nlme’ and ‘ggplot2’ [28].

## RESULTS

### Study population

**FIGURE 1:**
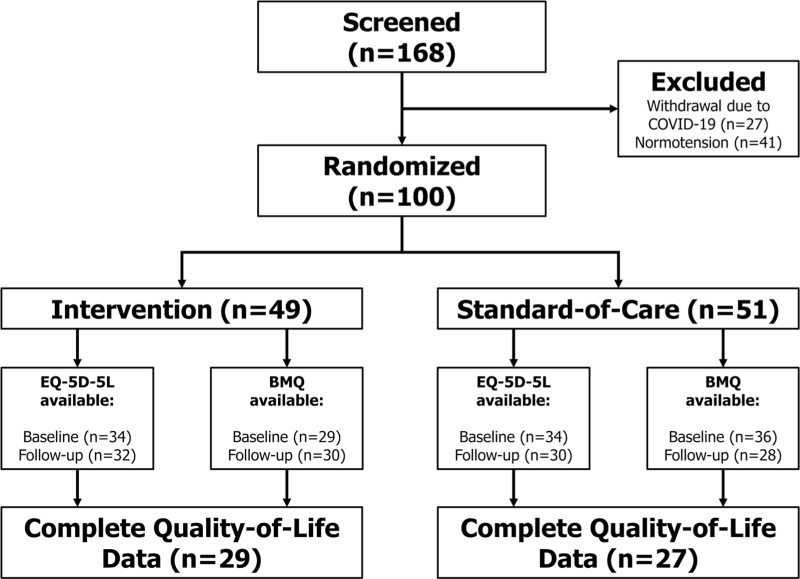
Study flowchart. BMQ, Beliefs about Medicine Questionnaire.

A total of 168 patients were screened and 141 completed a screening visit, consisting of a 24 h ambulatory BP measurement and therapy adherence testing. Of these, 41 patients were excluded due to normotension at the baseline visit, and 100 patients who met all study criteria were enrolled. A cohort of 56 (56.0%) patients (*n* = 29 intervention; *n* = 27 standard-of-care) who completed all questionnaires were included in the current study (Fig. 1). Within this cohort (*n* = 56), median age was 61.5 [55.8–69.3] years and 12 (21.4%) patients were women. Mean baseline BP was 149.8/84.1 ± 14.9/13.8 mmHg whereas patients were prescribed a median of 5.6 [4.8–7.3] defined daily dosages (DDD) of antihypertensive drugs. Baseline characteristics were distributed equally between both study arms (Table 1).

**TABLE 1:** Baseline characteristics

	Intervention + standard-of-care arm (*n* = 29)	Standard-of-care only arm (*n* = 27)	*P* value
Age (years) (mean ± SD)	63.8 ± 9.1	59.1 ± 11.6	0.10
Female sex [*n* (%)]	7 (24.1)	5 (18.5)	0.75
Body mass index (kg/m^2^) (median [25th–75th percentile]	28.4 [27.4-32.5]	28.5 [26.7–33.0]	0.90
Estimated glomerular filtration rate (ml/min/1.73 m^2^), median [25th–75th percentile]	61.0 [41.0–83.0]	81.0 [53.0–90.0]	0.07
Medical history			
Diabetes mellitus [*n* (%)]	11 (37.9)	14 (51.9)	0.42
Myocardial infarction [*n* (%)]	11 (37.9)	3 (11.1)	0.03
Stroke [*n* (%)]	4 (13.8)	2 (7.4)	0.67
Atrial fibrillation [*n* (%)]	4 (13.8)	3 (11.1)	1.00
Heart failure [*n* (%)]	0 (0.0)	3 (11.1)	0.11
Blood pressure			
Systolic blood pressure (mmHg), mean ± SD	151.9 ± 15.0	147.5 ± 14.6	0.27
Diastolic blood pressure (mmHg), mean ± SD	83.1 ± 13.4	85.3 ± 14.3	0.56
Drug prescriptions			
Total number of drugs prescribed, median [25th–75th percentile]	11.0 [7.0–12.0]	11.0 [6.5–12.5]	0.56
Number of antihypertensive drugs prescribed, median [25th–75th percentile]	4.0 [3.0–5.0]	4.0 [4.0–5.0]	0.23
DDDs of antihypertensive drugs prescribed, median [25th–75th percentile]	5.5 [4.5–7.2]	5.7 [4.8–7.3]	0.38
Antihypertensive drug adherence			
Complete adherence^a^ [*n* (%)]	20 (69.0)	19 (70.4)	0.51
Partial adherence^a^ [*n* (%)]	8 (27.6)	5 (18.5)	
Nonadherence^a^ [*n* (%)]	1 (3.4)	3 (11.1)	
Percentage of measurable drugs detected, mean ± SD/median [25th–75th percentile]	85.1 ± 25.6/100.0 [75.0–100.0]	81.2 ± 34.1/100.0 [75.0–100.0]	0.95

DDD, defined daily dosage; SD, standard deviation.

a Adherence to measurable drugs was categorized based on the percentage of drugs detected out of drugs prescribed: complete adherence (100%), partial adherence (in between 0 and 100%) or nonadherence (0%).

Within the whole RHYME-RCT cohort, questionnaire-responders (current study population) were older, more frequently men and prescribed a lower number of DDDs as compared with nonresponders (Supplementary Table 1). Study questionnaire data was not routinely collected in patients excluded at the eligibility visit (*n* = 41).

### EuroQol 5D-5L and Beliefs about Medicine Questionnaire outcomes

**FIGURE 2:**
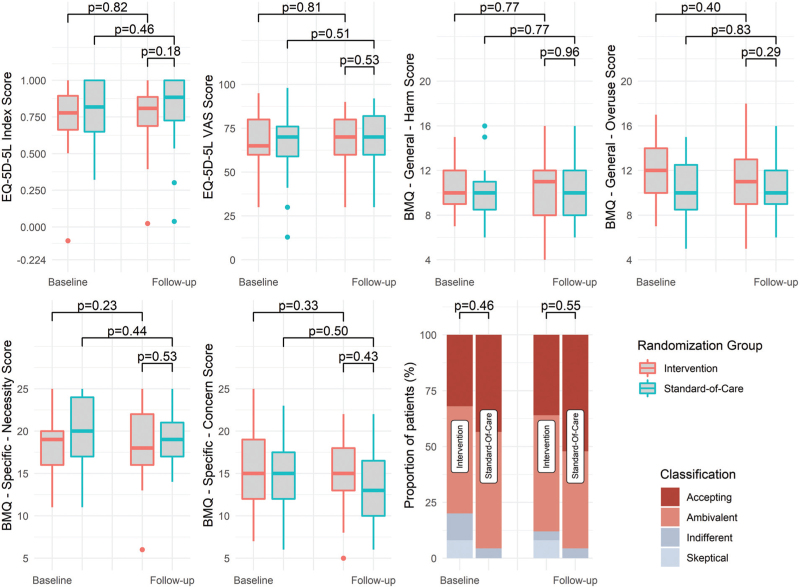
Changes in the five-level EuroQol 5D-5L (a) index score and (b) VAS score and Beliefs about Medicine Questionnaire (c) general harm, (d) general overuse (e) specific necessity, (f) specific concern scores and (g) specific attitude classification at baseline and 12 months post randomization. No between-group differences were detected for any of the scores at baseline.

Twelve months post randomization, no differences between the intervention arm and the standard-of-care arm were observed in median EQ-5D-5L index score (0.81 [0.69–0.89] vs. 0.89 [0.73–1.00]; *P* = 0.18) and median VAS score (70 [60–80] vs. 70 [60–82]; *P* = 0.53) (Fig. 2a and b). Likewise, no differences were observed between study arms with regard to the individual EQ-5D-5L dimensions of mobility, self-care, usual activities, pain/discomfort and anxiety/depression (Table 2). Furthermore, the intervention did not affect the BMQ-General scores on harm and overuse, nor the BMQ-Specific scores on necessity, concern or patient attitude at 12 months (Fig. 2c and g). Similar results were observed in a subgroup of patients who were partially adherent or nonadherent at baseline (*n* = 17; Supplementary Table 2 and Supplementary Figure 1).

**TABLE 2:** Distribution of the five-level EuroQol 5D-5L dimensions at baseline and 12-month follow-up

	Baseline		Follow-up		
Dimension	Intervention (*n* = 28)	Control (*n* = 23)	Intervention (*n* = 28)	Control (*n* = 23)	*P* value (12 months)
Mobility				
No problems	10 (35.7)	13 (56.5)	11 (39.3)	13 (56.5)	0.56
Slight problems	9 (32.1)	6 (26.1)	6 (21.4)	4 (17.4)	
Moderate problems	6 (21.4)	3 (13.0)	7 (25.0)	5 (21.7)	
Severe problems	3 (10.7)	1 (4.3)	4 (14.3)	1 (4.3)	
Unable to walk about	0 (0.0)	0 (0.0)	0 (0.0)	0 (0.0)	
Self-care				
No problems	26 (92.9)	20 (87.0)	26 (92.9)	20 (87.0)	0.32
Slight problems	2 (7.1)	1 (4.3)	1 (3.6)	3 (13.0)	
Moderate problems	0 (0.0)	1 (4.3)	0 (0.0)	0 (0.0)	
Severe problems	0 (0.0)	1 (4.3)	1 (3.6)	0 (0.0)	
Unable to wash or dress	0 (0.0)	0 (0.0)	0 (0.0)	0 (0.0)	
Usual activities				
No problems	14 (50.0)	13 (56.5)	12 (42.9)	13 (56.5)	0.83
Slight problems	7 (25.0)	4 (17.4)	10 (35.7)	6 (26.1)	
Moderate problems	5 (17.9)	2 (8.7)	3 (10.7)	3 (13.0)	
Severe problems	1 (3.6)	2 (8.7)	2 (7.1)	1 (4.3)	
Unable to do usual activities	1 (3.6)	2 (8.7)	1 (3.6)	0 (0.0)	
Pain/discomfort				
No pain or discomfort	7 (25.0)	9 (39.1)	8 (28.6)	11 (47.8)	0.71
Slight pain or discomfort	9 (32.1)	5 (21.7)	11 (39.3)	6 (26.1)	
Moderate pain or discomfort	8 (28.6)	8 (34.8)	7 (25.0)	4 (17.4)	
Severe pain or discomfort	4 (14.3)	0 (0.0)	1 (3.6)	1 (4.3)	
Extreme pain or discomfort	0 (0.0)	1 (4.3)	1 (3.6)	1 (4.3)	
Anxiety/depression				
Not anxious or depressed	22 (78.6)	15 (65.2)	22 (78.6)	17 (73.9)	0.98
Slightly anxious or depressed	5 (17.9)	4 (17.4)	3 (10.7)	3 (13.0)	
Moderately anxious or depressed	0 (0.0)	3 (13.0)	2 (7.1)	2 (8.7)	
Severely anxious or depressed	0 (0.0)	1 (4.3)	0 (0.0)	1 (4.3)	
Extremely anxious or depressed	1 (3.6)	0 (0.0)	1 (3.6)	0 (0.0)	

All values are represented as counts (percentages).

### Predictors of therapy adherence

Independent predictors of better therapy adherence throughout the course of the study were higher patient age [0.6 (95% CI 0.0–1.1) percent/year; *P* = 0.04], higher EQ-5D-5L index score [39.6 (12.7–66.5) percent/score-unit; *P* = 0.01], higher BMQ-specific necessity score [1.7 (0.2–3.3) percent/score-unit; *P* = 0.03] and higher BMQ-specific concern score [1.7 (0.1–3.2) percent/score-unit; *P* = 0.03]. In contrast, an increase in the total number of drugs prescribed was independently associated with a lower degree of therapy adherence [−1.5 (−2.6 to −0.4) percent/drug; *P* = 0.01; Table 3].

**TABLE 3:** Independent predictors of percentage adherence to antihypertensive drugs throughout the course of the study

	Effect estimate (%)	95% confidence interval	*P* value
Patient characteristics			
Age (years)	0.6	0.0–1.1	0.04
Female sex	7.6	−5.8 to 21.1	0.26
EQ-5D-5L (range)			
Index score (-0.22 to 1.00)	39.6	12.7–66.5	0.01
VAS score (0 to 100)	−0.1	−0.5 to 0.3	0.57
Beliefs about Medicine – General (range)			
Harm score (4–20)	−0.7	−3.3 to 1.9	0.58
Overuse score (4–20)	−1.3	−3.7 to 1.1	0.27
Beliefs about Medicine – Specific (range)			
Necessity score (5–25)	1.7	0.2–3.3	0.03
Concern score (5–25)	1.7	0.1–3.2	0.03
Drug prescriptions			
Total number of drugs	−1.5	−2.6 to −0.4	0.01

EQ-5D-5L, the five-level EuroQol 5D-5L.

## DISCUSSION

The current study is among the first randomized clinical trials on a personalized feedback conversation about therapy adherence on patient-oriented outcomes in patients with resistant hypertension. We were not able to demonstrate an effect of the intervention on HRQOL and patients’ beliefs about medication.

With regard to HRQOL, the intervention studied demonstrated neutral results. This could be explained by high baseline HRQOL scores, echoed by a prevalence of severe problems or disability below 20% for all HRQOL domains. This favorable level of HRQOL could be explained by the inclusion of more health-conscious patients, who were willing to participate in an interventional study for 1 year. Indeed, baseline HRQOL in our study was more favorable as compared with previously reported figures, reflected by a higher EQ-5D-5L index score (0.79 vs. 0.47, respectively) and VAS score (67 vs. 64, respectively) [18]. These differences could potentially explain the discrepancy in study outcomes, as a previous study in patients with less favorable HRQOL demonstrated a harmful effect of a therapy adherence intervention [18].

With respect to patients’ beliefs about medication, the adherence intervention did not show an improvement in subject-specific or general BMQ scores at 12 months. Similar to HRQOL, this could be explained by more positive beliefs about medication at baseline in the current study as compared with previous work [23]. These differences could also explain the discrepancy between our neutral findings and the positive effect of a therapy adherence intervention on the general harm score and the subject-specific concern score [23]. Ultimately, this could reflect a relationship between the baseline levels of patient-reported outcomes and the effect of therapy adherence interventions.

Within the entire RHYME-RCT study, BP significantly decreased in both arms while adherence improved only in the intervention arm [12]. Previous cross-sectional work indicated a positive relationship between BP and HRQOL and between therapy adherence and HRQOL [13,29]. Remarkably, improvements in BP and adherence were not coupled to a gain in HRQOL in our study. This observation emphasizes the complexity of such relationships, and might explain the conflicting results of therapy adherence interventions on HRQOL as reported in previous trials [16–18]. In resistant hypertension, which is usually an asymptomatic condition, a hypothetical, more pronounced effect of any BP-lowering intervention on HRQOL could be expected on the long-term, as by then more cardiovascular adverse events, which are associated with a detrimental effect on HRQOL, could have been prevented [5,30]. Therefore, future research should focus on long-term effects of therapy adherence interventions on HRQOL.

Within the study protocol, patients visited a dedicated hypertension outpatient clinic four times within a 12-month time period, while performing a similar number of 24 h ambulatory BP measurements [11]. Irrespective of the intervention, BP decreased by 12/6 mmHg within a 12-month time period [12]. Even in the absence of improvements in HRQOL or beliefs about medication, these results emphasize the potential value of a high-frequency contact between healthcare providers and patients with resistant hypertension. However, the absence of a comparator arm (involving less clinic visits and 24 h ambulatory BP measurements) precludes any statements on the efficacy of the current study protocol. Furthermore, the cardiovascular protective effect of intensive antihypertensive therapy diminishes after discontinuation of the intensive therapy regimen, emphasizing the importance of the durability of any BP-lowering intervention [31]. Within that context, we demonstrated that patients’ beliefs about the necessity of drugs and harmful drug effects remained suboptimal at the end of follow-up, questioning the durability of any changes in therapy adherence and BP. Future research should focus on the long-term efficacy of a high-frequency outpatient clinic visit schedule, including 24 h ambulatory BP measurements and adherence testing in resistant hypertension patients. Despite the current neutral results, patient-reported outcome measures could still be of use to identify patients in need of prolonged or repeat interventions in future studies.

Within the current study, previous findings on predictors of therapy adherence were replicated. As such, we identified higher patient age and a higher EQ-5D-5L index score as independent predictors of better therapy adherence [13,14,32]. In contrast to the EQ-5D-5L index score, the EQ-5D-5L VAS score was not a predictor of therapy adherence. This phenomenon emphasizes the importance of a multidomain questionnaire in the assessment of patients’ functional status, which is included in the index score but not in the VAS score. Future studies should provide more insights in any discrepancies between both scores which are derived from the same questionnaire. Furthermore, we identified BMQ-specific necessity and concern scores as significant predictors of therapy adherence, reflecting more favorable adherence in patients with stronger beliefs about the necessity of their medication as well as in those with more concerns about their medication, respectively. The latter could be explained by an increase in health-related concerns and anxiety leading to compensatory behavior, which in turn resulted in more favorable therapy adherence. For antihypertensive pharmacotherapy specifically, limited data is available on the relationship between BMQ-scores and therapy adherence, as previous studies included heterogeneous populations or relied on self-reported or pharmacy-reported adherence measures [19,33]. Before implementation in routine practice, the additive value of BMQ-scores in identification of nonadherent hypertensive patients should be validated in larger clinical studies using biochemically confirmed adherence measures. Finally, the total number of drugs prescribed was a predictor of therapy adherence, whereas the number of antihypertensive drugs was not. Whereas the total number of drugs is considered a relevant metric to measure drug burden on the patient level, previous work incorporated only antihypertensive drug burden, and this is a relevant strength (and an element of novelty) of the current study [34].

### Limitations

This study has several limitations. First, this study analyzed a prespecified secondary outcome of the RHYME-RCT, which was not statistically powered to detect a difference in HRQOL outcomes and which was halted prematurely [12]. Consequently, the current study could be statistically underpowered. Second, only 56% of the patients invited completed all questionnaires at baseline and follow-up. This selection probably resulted in a certain degree of selection bias for the EQ-5D-5L and BMQ outcomes in this open-label study. More specifically, we cannot rule out selective (non)response by patients based on their randomization arm. Furthermore, no questionnaire data was available for patients excluded prerandomization. Third, the generalizability of our conclusions on HRQOL and beliefs about medication should be treated with caution, as questionnaire respondents were significantly older, more often men and were prescribed a smaller number of antihypertensive drug DDDs as compared with nonrespondents. In addition, our findings should be interpreted while taking into account the prevalence of complete therapy adherence, which was higher in our study population as compared with a general population of resistant hypertension patients (69.6 vs. 52.1%, respectively) [35].

### Conclusions

In this study, we demonstrated that a personalized feedback conversation targeting therapy adherence does not improve HRQOL and beliefs about medication in patients with resistant hypertension. In parallel, we confirmed that higher age, better HRQOL, perceived necessity and concerns regarding antihypertensive drugs and a lower total number of drugs predicted better adherence to antihypertensive drugs. Despite the neutral findings on the intervention, future research on the topic should include patient-reported outcome measures.

## ACKNOWLEDGEMENTS

Funding: this study was supported by a ZonMW grant for Rational Pharmacotherapy [Project Number 848016003, Resistant HYpertension: MEasure to ReaCh Targets (RHYME-RCT)].

### Conflicts of interest

L.E.J.P. has received lecture fees from Astellas Pharma. L.v.D. has received research grants from Biogen and Teva Pharmaceuticals for studies not related to this study. J.D. received institutional grant/research support from ACIST Medical, Abbott Vascular, Boston Scientific, Medtronic, Microport, Pie Medical, and ReCor medical, and consultancy and speaker fees from Abbott Vascular, Abiomed, ACIST medical, Boston Scientific, Cardialysis BV, CardiacBooster, Kaminari Medical, Medtronic, PulseCath, Pie Medical, ReCor Medical, Sanofi and Siemens Healthcare. All other authors declare no competing interests.

## Supplementary Material

Supplemental Digital Content
