# Supplementary material for: Quality-of-life and beliefs about medication in relation to a therapy adherence intervention in resistant hypertension: the Resistant HYpertension: MEasure to ReaCh Targets trial
Source: J Hypertens. 2024 May 23;42(10):1687–94. doi: 10.1097/HJH.0000000000003780 (PMC11356742; doi:10.1097/HJH.0000000000003780)

**Supplementary Methods 1. Interpretation of Beliefs About Medicine (BMQ) questionnaire scores.**

General scores:

Increase in harm score (range 4-20): more harmful beliefs about medication effects.

Increase in overuse score (range 4-20): stronger beliefs about overuse of medication by physicians.

Specific scores:

Increase in necessity score (range 5-20): greater perceived necessity of the individual patient’s medication.

Increase in concern score (range 5-20): greater concerns about the effectivity of the individual patient’s medication.

Attitude classification based on specific scores:

Accepting: necessity score >16, concern score ≤13

Ambivalent: necessity score >16, concern score >13

Indifferent: necessity score ≤16, concern score ≤13

Skeptical: necessity score ≤16, concern score >13

**Supplementary Table 1. Comparison of quality-of-life and beliefs about medication questionnaires responders (current study population) vs. non-responders (not included in current study) within the whole RHYME-RCT study cohort.**

|  | **Total RHYME RCT population (n=100)** | **Questionnaire responders (n=56)*** | **Questionnaire non-responders (n=44)*** | **P-value** |
| --- | --- | --- | --- | --- |
| Age (years), median [25^th^-75^th^ percentile] | 61.0 [52.0-67.3] | 61.5 [55.8- 69.3] | 58.0 [48.8-65.5] | 0.04 |
| Female sex | 30 (30.0) | 12 (21.4) | 18 (40.9) | 0.048 |
| Body Mass Index (kg/m^2^), median [25^th^-75^th^ percentile] | 28.7 [26.7-33.2] | 28.5 [26.9-32.7] | 29.0 [26.5- 34.1] | 0.94 |
| Estimated glomerular filtration rate (ml/min/1.73m^2^), median [25^th^-75^th^ percentile] | 75.0 [48.0-90.0] | 69.5 [46.0-89.3] | 78.5 [55.3-90.0] | 0.28 |
| Medical history |  |  |  |  |
| Diabetes mellitus, n (%) | 37 (37.0) | 25 (44.6) | 12 (27.3) | 0.10 |
| Myocardial infarction, n (%) | 21 (21.0) | 14 (25.0) | 7 (15.9) | 0.32 |
| Stroke, n (%) | 11 (11.0) | 6 (10.7) | 5 (11.4) | 1.00 |
| Atrial fibrillation, n (%) | 9 (9.0) | 7 (12.5) | 2 (4.5) | 0.29 |
| Heart failure, n (%) | 4 (4.0) | 3 (5.4) | 1 (2.3) | 0.63 |
| Blood pressure |  |  |  |  |
| Systolic blood pressure (mmHg), mean ± SD | 151.1 ± 15.3 | 149.8 ± 14.9 | 152.7 ± 15.8 | 0.35 |
| Diastolic blood pressure (mmHg), mean ± SD | 85.7 ± 13.5 | 84.1 ± 13.8 | 87.7 ± 13.0 | 0.18 |
| Drug prescriptions |  |  |  |  |
| Total number of drugs prescribed, median [25^th^-75^th^ percentile] | 10.0 [6.5-12.0] | 11.0 [6.8-12.3] | 9 [6.5-12.0] | 0.42 |
| Number of antihypertensive drugs prescribed, median [25^th^-75^th^ percentile] | 4.0 [4.0-5.0] | 4.0 [3.8-5.0] | 4.0 [4.0-5.0] | 0.10 |
| DDDs of antihypertensive drugs prescribed, median [25^th^-75^th^ percentile] | 6.3 [4.8-7.3] | 5.6 [4.8-7.3] | 7.0 [5.3-8.0] | 0.02 |
| Antihypertensive drug adherence |  |  |  |  |
| Complete adherence, n (%) | 68 (68.0) | 39 (69.6) | 29 (65.9) | 0.33 |
| Partial adherence, n (%) | 21 (21.0) | 13 (23.2) | 8 (18.2) |  |
| Non-adherence, n (%) | 11 (11.0) | 4 (7.1) | 7 (15.9) |  |
| Percentage of measurable drugs detected, mean ± SD / median [25^th^-75^th^ percentile] | 80.2 ± 33.5 /  100 [75-100] | 83.2 ± 29.8 /  100 [75-100] | 76.4 ± 37.7 /  100 [73-100] | 0.53 |

* Availability of was defined as valid responses to the EQ-5D-5L and/or Beliefs About Medicine questionnaires at baseline and follow-up.

DDD, Defined Daily Dosage. SD, Standard Deviation.**Supplementary Table 2. Distribution of EQ-5D-5L dimensions at baseline and twelve-month follow-up in a subgroup of patients who were partially adherent or non-adherent at baseline (n=17).**

|  | **Baseline** | | **Follow-up** | | |
| --- | --- | --- | --- | --- | --- |
| **Dimension** | **Intervention (n=9)** | **Control (n=6)** | **Intervention (n=9)** | **Control (n=6)** | **P-value 12 months** |
| Mobility |  |  |  |  |  |
| No problems | 2 (22.2) | 4 (66.7) | 2 (22.2) | 4 (66.7) | 0.14 |
| Slight problems | 5 (55.6) | 1 (16.7) | 3 (33.3) | 0 (0.0) |  |
| Moderate problems | 2 (22.2) | 1 (16.7) | 4 (44.4) | 2 (33.3) |  |
| Severe problems | 0 (0.0) | 0 (0.0) | 0 (0.0) | 0 (0.0) |  |
| Unable to walk about | 0 (0.0) | 0 (0.0) | 0 (0.0) | 0 (0.0) |  |
| Self-care |  |  |  |  |  |
| No problems | 8 (88.9) | 5 (83.3) | 8 (88.9) | 5 (83.3) | 1.00 |
| Slight problems | 1 (11.1) | 0 (0.0) | 0 (0.0) | 1 (16.7) |  |
| Moderate problems | 0 (0.0) | 0 (0.0) | 0 (0.0) | 0 (0.0) |  |
| Severe problems | 0 (0.0) | 1 (16.7) | 1 (11.1) | 0 (0.0) |  |
| Unable to wash or dress | 0 (0.0) | 0 (0.0) | 0 (0.0) | 0 (0.0) |  |
| Usual activities |  |  |  |  |  |
| No problems | 5 (55.6) | 4 (66.7) | 3 (33.3) | 4 (66.7) | 0.69 |
| Slight problems | 2 (22.2) | 0 (0.0) | 4 (44.4) | 1 (16.7) |  |
| Moderate problems | 1 (11.1) | 0 (0.0) | 0 (0.0) | 1 (16.7) |  |
| Severe problems | 0 (0.0) | 2 (33.3) | 1 (11.1) | 0 (0.0) |  |
| Unable to do usual activities | 1 (11.1) | 0 (0.0) | 1 (11.1) | 0 (0.0) |  |

| Pain/discomfort |  |  |  |  |  |
| --- | --- | --- | --- | --- | --- |
| No pain or discomfort | 1 (11.1) | 2 (33.3) | 2 (22.2) | 2 (33.3) | 1.00 |
| Slight pain or discomfort | 3 (33.3) | 1 (16.7) | 3 (33.3) | 2 (33.3) |  |
| Moderate pain or discomfort | 1 (11.1) | 2 (33.3) | 3 (33.3) | 2 (33.3) |  |
| Severe pain or discomfort | 4 (44.4) | 0 (0.0) | 0 (0.0) | 0 (0.0) |  |
| Extreme pain or discomfort | 0 (0.0) | 1 (16.7) | 1 (11.1) | 0 (0.0) |  |
| Anxiety/depression |  |  |  |  |  |
| Not anxious or depressed | 7 (77.8) | 4 (66.7) | 7 (77.8) | 4 (66.7) | 0.72 |
| Slightly anxious or depressed | 1 (11.1) | 0 (0.0) | 1 (11.1) | 0 (0.0) |  |
| Moderately anxious or depressed | 0 (0.0) | 1 (16.7) | 0 (0.0) | 2 (33.3) |  |
| Severely anxious or depressed | 0 (0.0) | 1 (16.7) | 0 (0.0) | 0 (0.0) |  |
| Extremely anxious or depressed | 1 (11.1) | 0 (0.0) | 1 (11.1) | 0 (0.0) |  |

All values are represented as counts (percentages).

**Supplementary Figure 1. Changes in EQ-5D-5L (A.) index score and (B.) VAS score and Beliefs about Medicine Questionnaire (C.) general harm, (D.) general overuse, (E.) specific necessity, (F.) specific concern scores and (G.) specific attitude classification at baseline and twelve months post randomization in patients partially adherent or non-adherent at baseline**


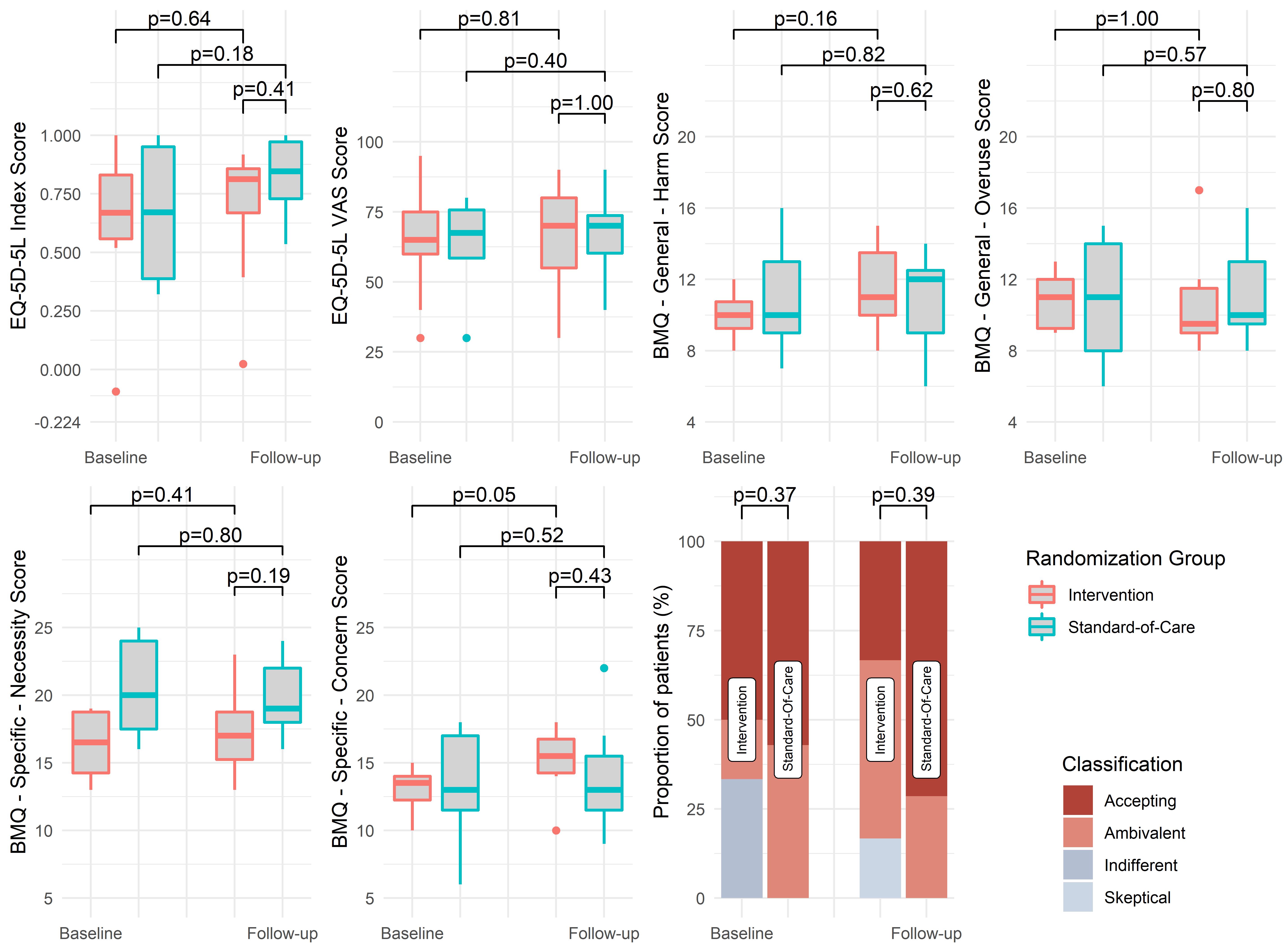

Supplement: Supplemental Digital Content [file jhype-42-1687-s001.docx]
